# Cerebral Hæmorrhage Due to Prolapsus Uteri

**Published:** 1907-07-06

**Authors:** 


### Points in Pathology.

### CEREBRAL HÆMORRHAGE DUE TO PROLAPSUS UTERI.

It is sometimes forgotten that the renal fibrosis caused by chronic ascending nephritis may produce results precisely similar to those of an ordinary granular kidney. Chronic ascending nephritis is very insidious in its onset; it results gradually from any condition in which there is obstruction to the outflow of urine from the ureters. It is a lesion quite distinct from the so-called surgical kidney, in which the changes are also "ascending"; in the surgical kidney proper the ascending nephritis is suppurative, and the symptoms are acute; in chronic ascending nephritis there is no suppuration, but a gradual replacement of the kidney substance by fibrous tissue until ultimately a deeply scarred and puckered viscus results, with diminished cortex, and an obviously granular surface when the capsule is peeled off.

It seems at first sight a far cry to connect a prolapsed uterus with a cerebral hæmorrhage, as cause and effect; but we have seen more than one example of the connection. The prolapsed uterus carries with it the bladder and the lower ends of the ureters; the latter become bent upon themselves, or kinked, at a point about one inch above their openings into the bladder. The result is an obstruction to the outflow of urine through the ureters, and this obstruction is occasionally sufficient to cause insidious but progressive fibrotic nephritis. The result of the renal degeneration is then similar to that of primary granular kidney; the left ventricle enlarges, the arteries degenerate, and the blood-pressure becomes high. Amongst the degenerate vessels may be those in the brain; the high blood-pressure ruptures one of these at its weakest spot, and the patient has a cerebral hæmorrhage which she probably would not have had if she had not suffered from a prolapsed uterus of long standing.

Fortunately this sequence of events is not common, probably because the ureters manage to accommodate themselves to their altered position without becoming greatly obstructed in most cases. Nevertheless, the undoubted occurrence of fibrotic kidneys from this cause, and the occasional termination of the case in apoplexy, must be an additional reason for doing everything possible for the relief of a prolapsed uterus.

